# Supplementary material for: The tardigrade Hypsibius exemplaris has the active mitochondrial alternative oxidase that could be studied at animal organismal level
Source: PLoS One. 2021 Aug 23;16(8):e0244260. doi: 10.1371/journal.pone.0244260 (PMC8382173; doi:10.1371/journal.pone.0244260)

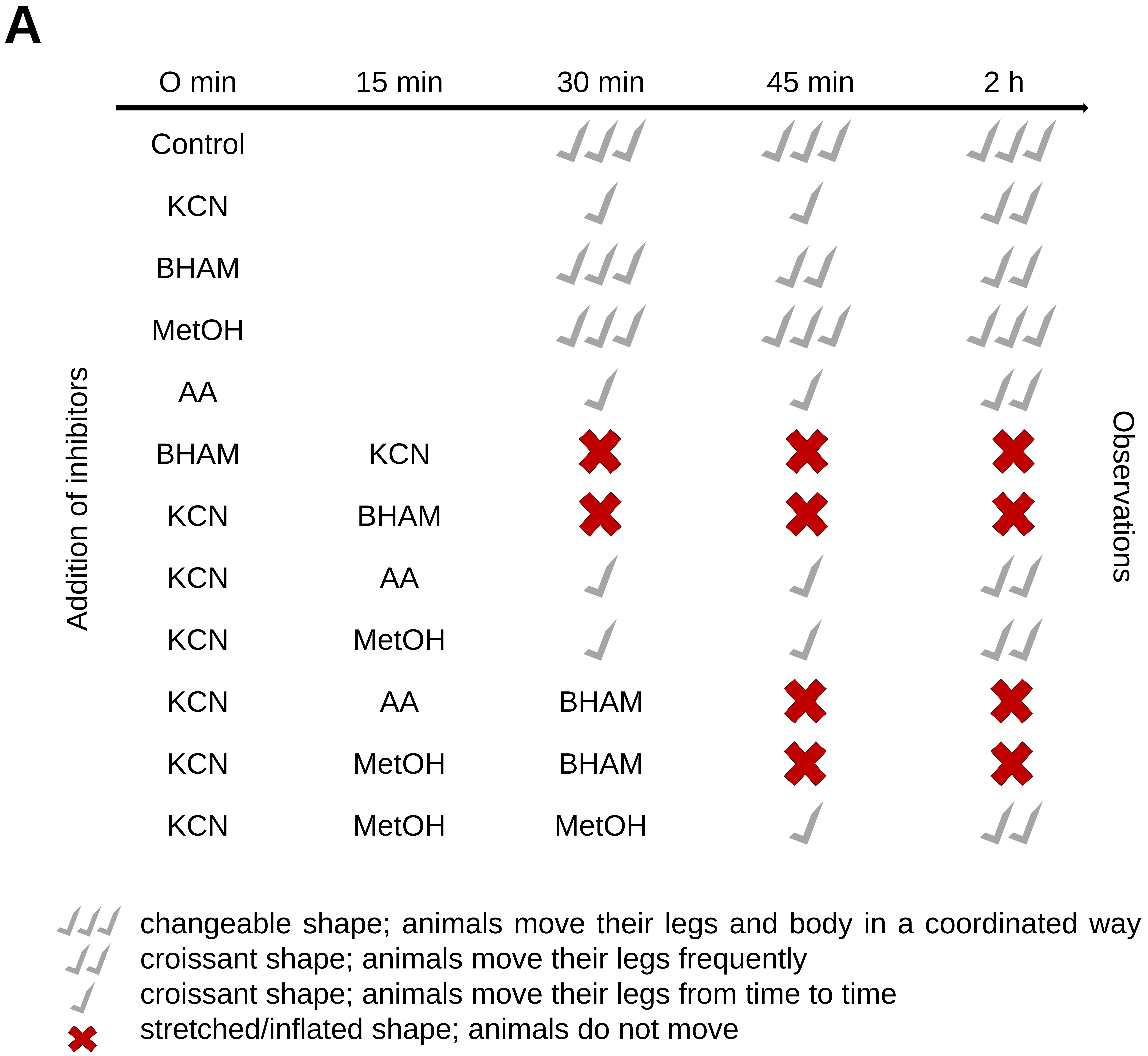
A
O min
15 min
30 min
45 min
2 h
Addition of inhibitors
Observations
Control
KCN
BHAM
MetOH
AA
BHAM KCN
KCN BHAM
KCN AA
KCN MetOH
KCN AA BHAM
KCN MetOH BHAM
KCN MetOH MetOH
changeable shape; animals move their legs and body in a coordinated way
croissant shape; animals move their legs frequently
croissant shape; animals move their legs from time to time
stretched/inflated shape; animals do not move

B

Control
30 min/2 h

KCN
30 min/2 h

BHAM
30 min/2 h

MetOH
30 min/2 h

AA
30 min/2 h

BHAM – KCN
30 min/2 h

KCN – BHAM
30 min/2 h

KCN – AA
30 min/2 h

KCN – MetOH
30 min/2 h

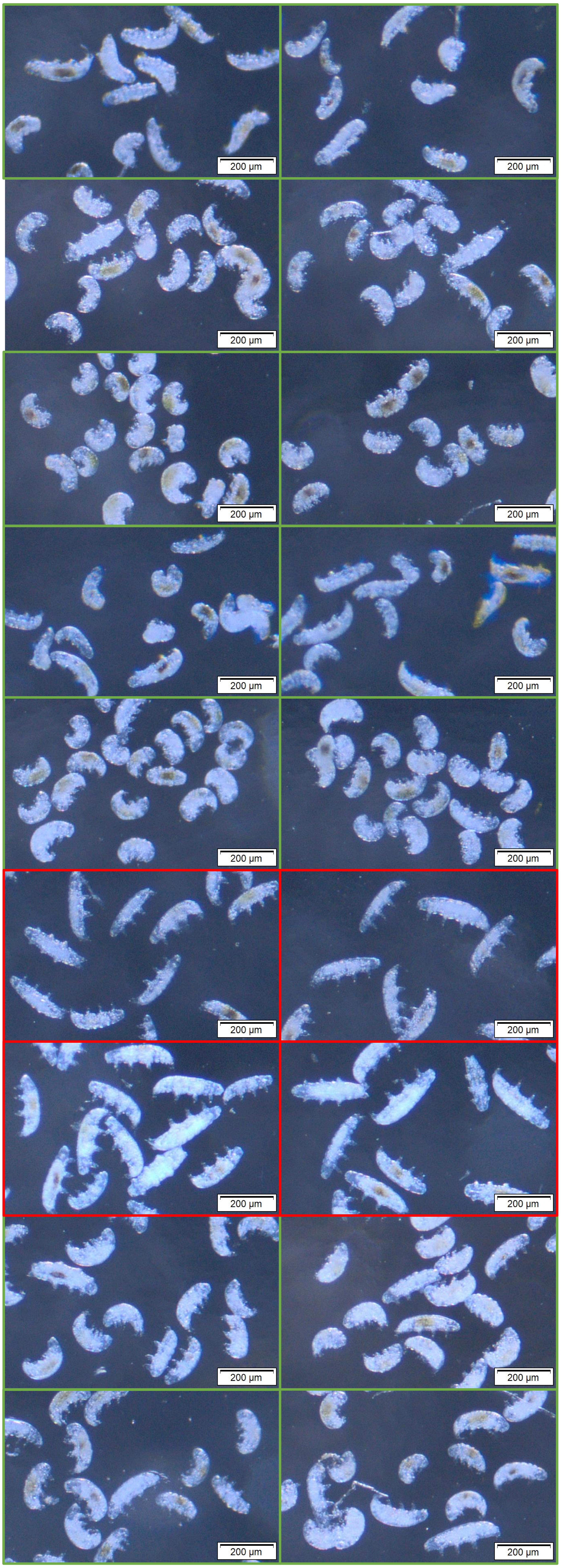

KCN – AA – BHAM
45 min/2 h

KCN – MetOH – BHAM
45 min/2 h

KCN – MetOH – MetOH
45 min/2 h

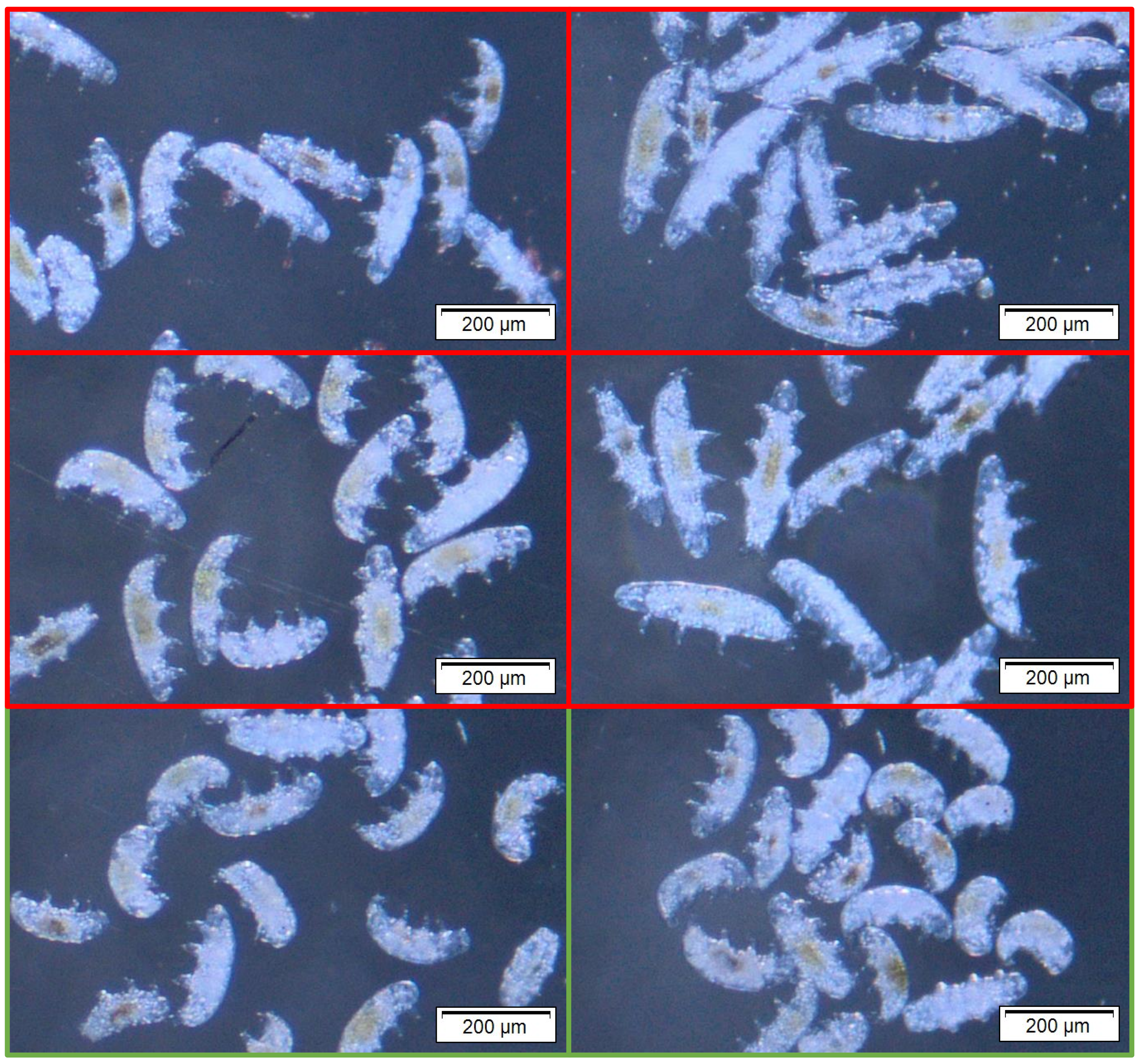

Supplement: S1 Fig — The data represent two independent repeats of the test and each tested group consisted of 20 specimens. The images present all variants of the performed test. (PDF) [file pone.0244260.s001.pdf]
