# Supplementary material for: The tardigrade Hypsibius exemplaris has the active mitochondrial alternative oxidase that could be studied at animal organismal level
Source: PLoS One. 2021 Aug 23;16(8):e0244260. doi: 10.1371/journal.pone.0244260 (PMC8382173; doi:10.1371/journal.pone.0244260)
Supplement: S1 Table — (DOCX) [file pone.0244260.s002.docx]

Table S1. Oxygen consumption rates corresponding to mitochondria-based respiration of intact specimens in the presence of the MRC complex III and IV inhibitors (AA and KCN, respectively) and AOX inhibitor (BHAM). The data in each row are from the same experiment and represent calculations made for individual traces obtained in the presence and in the absence of animals. The latter serve as a control for oxygen consumption. 10 000 animals were used for one trace and the presented values of the oxygen consumption rate are calculated for 10 000 animals. The values reported for KCN, AA, and BHAM columns were obtained for independent experiments, represent values calculated for individual traces and do not reflect sequential additions. A. KCN ± AA added prior to BHAM; B. BHAM added before KCN. Basal respiration denotes oxygen consumption rate recorded in the presence of animals.

|  |  | O_2_ consumption rate [nMO_2_/min] | | | |  | O_2_ consumption rate [nMO_2_/min] | | | |
| --- | --- | --- | --- | --- | --- | --- | --- | --- | --- | --- |
| A | Trace number | animals | KCN | AA | BHAM | A | no animals | KCN | AA | BHAM |
|  | 1 | 4.78 | 2.32 |  | 0.67 |  | 0.66 | 0.81 |  | 0.79 |
|  | 2 | 3.64 | 1.78 |  | 0.47 |  | 0.85 | 0.34 |  | 0.09 |
|  | 3 | 5.5 | 2.96 | 3.02 | 0 |  | 0.32 | 0.41 | 0.17 | 0.37 |
|  | 4 | 4.79 | 2.81 | 2.78 | 1.61 |  | 0.36 | 0.67 | 0.67 | 0.48 |
|  | 5 | 5.74 | 3.42 | 2.77 | 0 |  | 0.61 | 0.91 | 0.84 | 0.18 |
| B |  | animals | BHAM | KCN |  | B | no animals | BHAM | KCN |  |
|  | 1 | 4.31 | 4.56 | 0.94 |  |  | 0.39 | 0.56 | 0.93 |  |
|  | 2 | 7.7 | 7.36 | 0.75 |  |  | 0.23 | 0.29 | 0.56 |  |
|  | 3 | 3.19 | 2.77 | 0.69 |  |  | 0.53 | 0.49 | 0.38 |  |
