# Supplementary material for: The tardigrade Hypsibius exemplaris has the active mitochondrial alternative oxidase that could be studied at animal organismal level
Source: PLoS One. 2021 Aug 23;16(8):e0244260. doi: 10.1371/journal.pone.0244260 (PMC8382173; doi:10.1371/journal.pone.0244260)

**Table S2.** LC–MS/MS based detection of *H. exemplaris* AOX protein.

| Accession | Description | Coverage [%] | # PSMs | # Unique Peptides | # Amino acids | MW [kDa] | calc. pI | Abundance |
| --- | --- | --- | --- | --- | --- | --- | --- | --- |
| OWA52662.1 | Alternative oxidase, mitochondrial  [*Hypsibius exemplaris*] | 14 | 5 | 4 | 361 | 41.3 | 8.48 | 5274690.875 |

| Annotated Sequence | # Missed Cleavages | Theo.MH+ [Da] | Positions in Protein |
| --- | --- | --- | --- |
| [K].VDWLAYIAVQTLR.[K] | 0 | 1547,85804 | [160-172] |
| [K].SFDLISGYTFGR.[K] | 0 | 1362,66884 | [174-185] |
| [R].DHGWIHTLLEEAENER.[M] | 0 | 1948,91478 | [224-239] |
| [R].FVGYLEEEAVK.[T] | 0 | 1283,65179 | [284-294] |

Comparison of *H. exemplaris* AOX predicted amino acid sequence (OWA52662.1) with the sequence of *Ciona intestinalis*. The identified peptides are marked with color boxes. The conserved glutamate (E) and histidine (H) residues within the ferritin-like domain (black frame) are marked with stars. The extra sequence in the C-terminus of *H. exemplaris* AOX is a misannotation artifact (K. Arakawa personal communication).


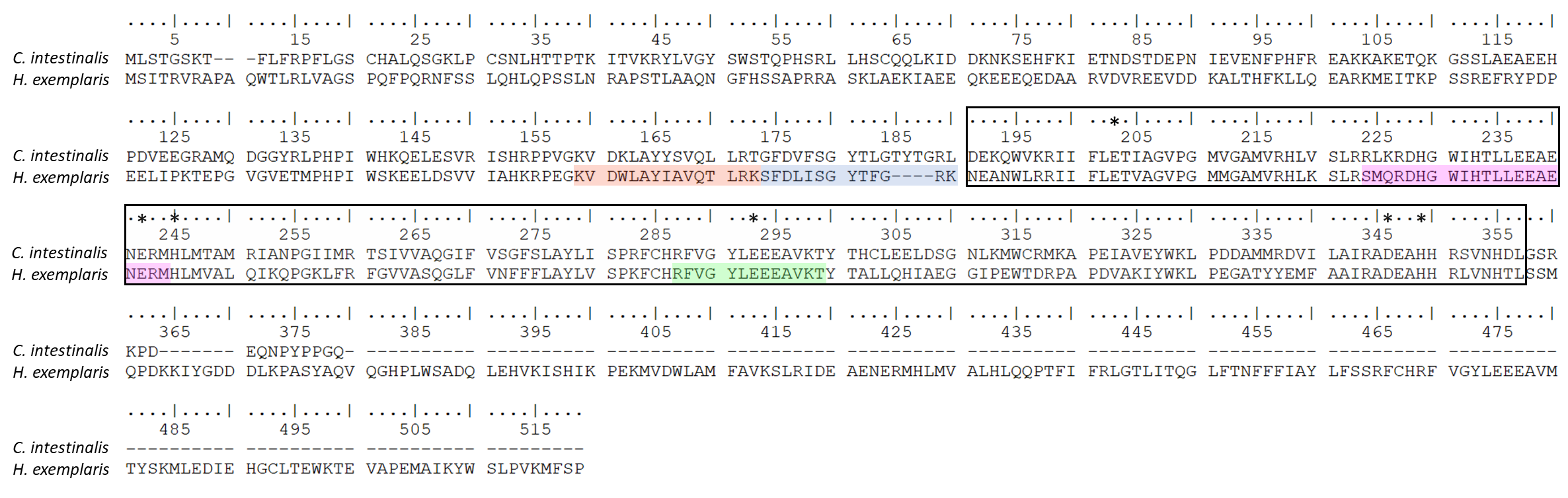

Supplement: S2 Table — The sequence alignment was obtained by ClustalW Multiple Alignment tool [46], implemented in BioEdit. The identified peptides are marked with color boxes. The conserved glutamate (E) and histidine (H) residues within the ferritin-like domain (black frame) are marked with stars. (DOCX) [file pone.0244260.s003.docx]
