# Supplementary material for: The tardigrade Hypsibius exemplaris has the active mitochondrial alternative oxidase that could be studied at animal organismal level
Source: PLoS One. 2021 Aug 23;16(8):e0244260. doi: 10.1371/journal.pone.0244260 (PMC8382173; doi:10.1371/journal.pone.0244260)
Supplement: Attachment — Submitted filename: PONE-D-20-38224 review.docx [file pone.0244260.s005.docx]

The paper of Grobys et al., “The tardigrade Hypsibius exemplaris as an emerging model to study the mitochondrial alternative oxidase at animal organismal level” demonstrates functionality of recently revealed ubiquinol oxidase in tardigrade species Hypsibius exemplaris. The work could be especially interesting for comparative physiologists and biochemists that focus on alternative respiratory pathways in animals and the role of these pathways in animal adaptation to functional hypoxia, respiratory chain inhibitors, temperature changes, and other stressors.

The work is quite laconic, however the results presented seem trustable and complete according to the authors’ intentions.

I do not have serious concerns regarding the experimental design and methodology as well as data representation. However, it is worth noting a number of minor concerns that should be taken into account to improve the reading and understanding of the authors’ introduction, data, and discussion:

Abstract, line 16:

The authors state “(AOX) is present in mitochondria of many invertebrates”. This sounds too general. In my opinion, it would be better to state “several invertebrate taxa” instead of “many invertebrates”. So far, alternative oxidase (AOX) was revealed in genomes of some sea squirts, crustaceans, mollusks, annelids, cnidarians, and sponges but not insects. It is not known so far whether occurrence of AOX in genomes of the listed invertebrate taxa is due to horizontal gene transfer. If the gene that encodes AOX was horizontally transferred from symbiotic fungi, plants or protists to the animals, then likely not all representatives of a particular taxon may contain it.

Abstract, line 21: The abbreviation MRC is not deciphered. It is present in the List of abbreviations, however direct introduction of this abbreviation in the Abstract would be desirable.

Abstract, lines 26-28: The authors did not study AOX expression (e.g. mRNA levels or detection by western blot, or enzymatic activity with its native substrate, ubiquinol) therefore it is not relevant to write that it was monitored.

Keywords, line 31:

I would suggest substitution the term “intact organism” with something like “whole-body respiration” or “whole organism/animal respiration”, as well as the term “KCN tolerance” with “cyanide resistance”.

Introduction, line 49: “understood” instead of “understand”

Introduction, line 77: In my opinion, there is no need to take additional reference for defining Drosophila melanogaster species since the organism is well-known. The same concern is about references 30, 31, and 33. They contain narrow taxonomical data and do not have relation to the main theme of the article which is considered to fall in the field of comparative physiology.

Introduction, lines 80-82: There should be a reference at the end of the sentence.

Introduction, line 89: I suggest “oxidant sensitivity” instead of “oxidant-sensitivity”.

Introduction, lines 96-98: The sentence is irrelevant. The study of Tward et al. (2019) criticized by the authors does not represent a functional study of the animal AOX but is rather a detection of AOX in the copepode Tigriopus californicus. The study of Tward et al. (2019) suggests the molecular mass of putative AOX basing on the preliminary immunobloting analysis but does not conclusively postulate it. The authors state “... its predicted amino acid sequence does not contain the proper C-terminus motif [34]”. What do they mean under “proper” C-terminus motif? Tward and colleagues have found C-terminal motif NPFEKGK of AOX from T. californicus which is indeed similar to the C-terminal motif of AOXs from other animals, e.g. Amphimedon queenslandica (NPFEPGK), Ciona intestinalis and Phallusia mammillata (NPYPPGQ), Branchiostoma floridae (NPFQPGK), and many others.

Introduction, lines 109-111: The references 37-40 do not have relation to the AOX genes or sequencing projects, therefore are misleading in relation to the context.

Introduction, line 118: Here, the authors use the term “mitochondrial respiratory system” whereas “mitochondrial respiratory chain” is in the abstract. I would suggest to either unify terminology throughout the text or explain the difference between the chain and the system.

Results, lines 165-169, and Figure 3:

The figure would benefit if contained respiration rates near the appropriate segments of the oxygen consumption curve. The marks of statistical significance should be shown on the Figure 3B.

Methods, line 253: A reference to the mineral content of the water would be helpful.

Methods, lines 272-275 or legends for Figures 2, and S1, and Figures 2, and S1:

The magnification of microscope (in the Materials and Methods and/or Figure legends) and scale bars in the corresponding figures would improve perception of the figures for readers.

Methods, lines 300-301: Formally, the abbreviation SDS-PAGE should be deciphered (despite it is widely used and well-known).
