# Supplementary material for: The tardigrade Hypsibius exemplaris has the active mitochondrial alternative oxidase that could be studied at animal organismal level
Source: PLoS One. 2021 Aug 23;16(8):e0244260. doi: 10.1371/journal.pone.0244260 (PMC8382173; doi:10.1371/journal.pone.0244260)
Supplement: Attachment — Submitted filename: Responses for reviewers.docx [file pone.0244260.s006.docx]

Dear Editor,

Thank you very much for positive comments concerning our manuscript entitled “The tardigrade *Hypsibius exemplaris* as an emerging model to study the mitochondrial alternative oxidase at animal organismal level” (PONE-D-20-38224) and the possibility to submit the revised version of the manuscript. We have read and discussed carefully all provided comments and tried to improve the manuscript accordingly to all the comments. The detailed description of the performed corrections (marked in red) is provided below:

Editor:

Personally, I find this study very interesting. However, the data presented is very limiting to assess the impact of AOX on overall mitochondrial bioenergetics of H. exemplaris. I would prefer to see the following:

1. The effects of different inhibitors including BHAM and other AOX inhibitors (if any, prolyl gallate?) in the presence and absence of AA and KCN on ATP and mitochondrial membrane potential.

Answer: We do not understand why we have to prove that BHAM is a specific inhibitor of AOX. It is well known that AOX activity is inhibited by primary hydroxamic acids such as BHAM. The inhibitor was repeatedly used in the study of AOX proteins of microorganisms and plants by one of the co-authors of the manuscript (Prof. Wieslawa Jarmuszkiewicz; e.g. doi: 10.1104/pp.119.4.1323, 10.1023/a:1022574327117, 10.1016/s0014-5793(02)03734-1, 10.1023/a:1023750204310, 10.1016/j.febslet.2005.04.081, 10.1016/j.bbabio.2015.01.005, 10.1016/j.redox.2018.07.018). Moreover, a broad range of available AOX inhibitors is designed to interact with the AOX diiron centre (e.g. doi:10.1002/med.21560). This also applies to BHAM and the most commonly used SHAM and n-propyl gallate. Therefore we expect comparable results in the presence of other inhibitors but of course the inhibitors display different K_i_ and IC_50_ values for AOX inhibition.

Unfortunately, access to laboratories is currently severely limited due to restrictions imposed by COVID-19. This causes reduction of the performed laboratory cultures that makes research on ATP synthesis impossible as it requires isolated mitochondria and series of experiments. The main reason is the amount of organisms that would have to be collected to obtain proper quantity of mitochondria to perform functional experiments. However, we were able to perform the inner mitochondrial membrane potential analysis for intact specimens using tetramethylrhodamine methyl ester (TMRM). This is a cell-permeant, cationic, fluorescent dye that is commonly applied in detection of the potential. The dye accumulates in hyperpolarised mitochondria and the fluorescence intensity of this compound correlates with the inner membrane potential. The results were added to the revised manuscript.

2. Molecular identification and characterization of the AOX protein/gene from H. exemplaris.

Answer: We did not concentrate on that issue as the work has already been done by Bemm et al 2017 (bioRxiv. 2017; 122309. DOI: 10.1101/122309) but we decided to add comparison of our mass spectrometry data with the *Ciona intestinalis* AOX sequence (Additional file 4).

3. Modification of the text as suggested by the reviewers.

Answer: We modified the text at the basis of Reviewers suggestions. If a given part was not changed we provided explanation.

Reviewer #1:

The paper of Grobys et al., “The tardigrade Hypsibius exemplaris as an emerging model to study the mitochondrial alternative oxidase at animal organismal level” demonstrates functionality of recently revealed ubiquinol oxidase in tardigrade species Hypsibius exemplaris. The work could be especially interesting for comparative physiologists and biochemists that focus on alternative respiratory pathways in animals and the role of these pathways in animal adaptation to functional hypoxia, respiratory chain inhibitors, temperature changes, and other stressors.

The work is quite laconic, however the results presented seem trustable and complete according to the authors’ intentions.

Answer: Thank you very much for the positive opinion about our work

I do not have serious concerns regarding the experimental design and methodology as well as data representation. However, it is worth noting a number of minor concerns that should be taken into account to improve the reading and understanding of the authors’ introduction, data, and discussion:

Answer: The indicated corrections have been introduced.

Abstract, line 16: The authors state “(AOX) is present in mitochondria of many invertebrates”. This sounds too general. In my opinion, it would be better to state “several invertebrate taxa” instead of “many invertebrates”. So far, alternative oxidase (AOX) was revealed in genomes of some sea squirts, crustaceans, mollusks, annelids, cnidarians, and sponges but not insects. It is not known so far whether occurrence of AOX in genomes of the listed invertebrate taxa is due to horizontal gene transfer. If the gene that encodes AOX was horizontally transferred from symbiotic fungi, plants or protists to the animals, then likely not all representatives of a particular taxon may contain it.

Answer: Corrected as suggested.

Abstract, line 21: The abbreviation MRC is not deciphered. It is present in the List of abbreviations, however direct introduction of this abbreviation in the Abstract would be desirable.

Answer: Corrected as suggested.

Abstract, lines 26-28: The authors did not study AOX expression (e.g. mRNA levels or detection by western blot, or enzymatic activity with its native substrate, ubiquinol) therefore it is not relevant to write that it was monitored.

Answer: We used the term “AOX expression” because we wanted to address the protein detection by MS analysis. But we agree, the term “AOX expression” was not very well selected. Therefore, we changed the mentioned sentence as follows: “Furthermore, it is also possible to check the impact of the MRC cytochrome pathway blockage on AOX level as well as AOX inhibition in the absence of the blockage on animal functioning”.

Keywords, line 31: I would suggest substitution the term “intact organism” with something like “whole-body respiration” or “whole organism/animal respiration”, as well as the term “KCN tolerance” with “cyanide resistance”.

Answer: Corrected as suggested.

Introduction, line 49: “understood” instead of “understand”

Answer: Of course, it should be “understood”

Introduction, line 77: In my opinion, there is no need to take additional reference for defining Drosophila melanogaster species since the organism is well-known. The same concern is about references 30, 31, and 33. They contain narrow taxonomical data and do not have relation to the main theme of the article which is considered to fall in the field of comparative physiology.

Answer: We removed all the taxonomic references.

Introduction, lines 80-82: There should be a reference at the end of the sentence.

Answer: The references have been added.

Introduction, line 89: I suggest “oxidant sensitivity” instead of “oxidant-sensitivity”.

Answer: Corrected as suggested.

Introduction, lines 96-98: The sentence is irrelevant. The study of Tward et al. (2019) criticized by the authors does not represent a functional study of the animal AOX but is rather a detection of AOX in the copepode Tigriopus californicus. The study of Tward et al. (2019) suggests the molecular mass of putative AOX basing on the preliminary immunobloting analysis but does not conclusively postulate it. The authors state “... its predicted amino acid sequence does not contain the proper C-terminus motif [34]”. What do they mean under “proper” C-terminus motif? Tward and colleagues have found C-terminal motif NPFEKGK of AOX from T. californicus which is indeed similar to the C-terminal motif of AOXs from other animals, e.g. Amphimedon queenslandica (NPFEPGK), Ciona intestinalis and Phallusia mammillata (NPYPPGQ), Branchiostoma floridae (NPFQPGK), and many others.

Answer: We are very ashamed of the mistake. The sentence was removed.

Introduction, lines 109-111: The references 37-40 do not have relation to the AOX genes or sequencing

Answer: We removed all the taxonomic references.

Introduction, line 118: Here, the authors use the term “mitochondrial respiratory system” whereas “mitochondrial respiratory chain” is in the abstract. I would suggest to either unify terminology throughout the text or explain the difference between the chain and the system.

Answer: We agree it should be unified so we replaced “mitochondrial respiratory system” by abbreviation of mitochondrial respiratory chain; i.e. ”MRC”.

Results, lines 165-169, and Figure 3: The figure would benefit if contained respiration rates near the appropriate segments of the oxygen consumption curve. The marks of statistical significance should be shown on the Figure 3B.

We changed Figure 3 taking into account comments of both the Reviewers.

Methods, line 253: A reference to the mineral content of the water would be helpful.

Answer: The proper reference is not available so we added information provided by the producer.

Methods, lines 272-275 or legends for Figures 2, and S1, and Figures 2, and S1:

The magnification of microscope (in the Materials and Methods and/or Figure legends) and scale bars in the corresponding figures would improve perception of the figures for readers.

Answer: The information concerning the microscope magnification was added to the Material and Methods section (Toxicity in vivo test).

Methods, lines 300-301: Formally, the abbreviation SDS-PAGE should be deciphered (despite it is widely used and well-known).

Answer: The correction has been introduced.

Reviewer #2: This paper tries to demonstrate a functional relationship between mitochondrial respiratory chain (MRC) and alternative oxidase (AOX) in the tardigrade Hypsibius exemplaris. Based on the measurements of respiration in the presence and absence of pharmacological inhibitors of the MRC and AOX, it proposes that H. exemplaris can serve as model for the study of mitochondrial AOX. While the presented data indeed indicates the possibility of H. exemplaris being a potential model organism for the study of AOX and its relationship with MRC in whole body physiology, the presented data is insufficient. There are several issues that must be clearly addressed.

Answer: Thank you very much for all comments allowing improvement of our manuscript. Taking the comments into account we decided to change the title of our manuscript as follows: “The tardigrade *Hypsibius exemplaris* has the active mitochondrial alternative oxidase that could be studied at animal organismal level” as well as introduced proper changes into Abstract. We also carefully considered all comments and introduced relevant modifications into the manuscript text.

1. Describe the criteria for selecting an animal model to study AOX and why H. exemplaris fits these criteria. Just BHAM sensitivity is does not make it a good model to study AOX. Other biological and genetic features must be described and how it can be used to model the consequences of mitochondrial dysfunction and therapeutic strategies for mitochondrial dysfunction in details.

a. It will be good to provide some information about how fast animals double (i.e. reproduction cycle). Otherwise if the animals become limiting, then this model is no good for experimental purposes.

Answer: The information about how fast the animals double has been added to the Materials and Methods section. We also added to the proper part of the section reference to our recently published paper concerning protocols of tardigrade culturing including *H. exemplaris*.

Also some information on their genetics and potential genetic tractability and its utility to model human disease/aging will be useful.

Answer: We addressed the suggestion together with other issues mentioned in p. 1 in the Discussion section. Nevertheless, we have to mention that we did not intend to present *H. expemplaris* as a fully described model for animal AOX studies. We only indicated that the animal may be considered as such a model due to the presence of native AOX and easy respiration monitoring and behavioural observations at the organismal level. That is why we used “emerging model” in the title. In the revised manuscript respiration and behavioural data were supplemented with the mitochondrial inner membrane detection in intact specimens by application of TMRM staining.

2. Figure 1: It would be good to show the stoichiometry of H+ pumped out with and without AOX operating and discuss its impact on the ATP yield.

**Answer:** The stoichiometry of H+/ATP (ATP synthesis yield) concerns the mitochondrial respiratory chain complexes. We might of course discuss the effect of AOX activation on ATP synthesis, taking into account differences in the respiratory chain organization and the enzyme activation mechanism observed for plant and non-plant mitochondria. However, in the case of animals the data concerns heterologously expressed AOX and as mentioned above at present we are not able to provide data on *H. exemplaris* isolated mitochondria.

3. Apart from respiration assay, no attempt was made to understand the impact of AOX and MRC inhibitions on ATP and mitochondrial membrane potential. Measurements of ATP and membrane potential do not require species-specific reagents such as antibody. Therefore, ATP and mitochondrial membrane potential data can be easily obtained using standard procedures with minimal optimizations. Such data are essential to understand the role of AOX in mitochondrial bioenergetics.

Answer: We agree with the Reviewer. Some of us work in the Department of Bioenergetics and we know the methods suggested by the Reviewer. But they require application of isolated mitochondria. Unfortunately, at present studies on isolated mitochondria of *H. exemplaris* are impossible due to distinctly reduced culture of the organism being a consequence of COVID-19 restrictions. However, we were able to perform the inner mitochondrial membrane analysis for intact specimens using tetramethylrhodamine methyl ester (TMRM). This is a cell-permeant, cationic, fluorescent dye that is commonly applied in detection of the potential. The dye accumulates in hyperpolarised mitochondria and the fluorescence intensity of this compound correlates with the inner membrane potential. The data were added to the revised manuscript. We also addressed the mentioned issues in the Discussion section.

4. Evidence for the BHAM sensitivity and its specificity to AOX of H. exemplaris should be provided. BAHM sensitivity and mass spectrometry do not provide direct evidence for AOX function. One reference cited as evidence for the specificity of BHAM for AOX appears to be a review article.

Answer: We cannot agree. We used the review paper as the reference to make the citation more general instead of a bundle of experimental papers involving BHAM application as AOX inhibitor. For example, the inhibitor was repeatedly used in the study of AOX proteins of microorganisms and plants by one of the co-authors of the manuscript (Prof. Wieslawa Jarmuszkiewicz; e.g. doi: 10.1104/pp.119.4.1323, 10.1023/a:1022574327117, 10.1016/s0014-5793(02)03734-1, 10.1023/a:1023750204310, 10.1016/j.febslet.2005.04.081, 10.1016/j.bbabio.2015.01.005, 10.1016/j.redox.2018.07.018). Moreover, in our recently published paper we proved that the tardigrade *Milnesium inceptum* AOX is inhibited by BHAM (doi: 10.1186/s12983-021-00400-5). We added the observation and reference to the proper part of the Discussion section.

5. Figure 2 is highly confusing. Both figure legends and method sections do not provide enough details of the experiments.

a. All data should be reported in quantitative terms by giving the % animals responding to inhibitor(s) at each time (or one chosen time point). For example % animals mobile or immobile and their shapes at each time point.

b. Data from Figueres 2 and S1 can be combined in one figure. Only the video file should be presented as a supplement.

**Answer:** We considered quantitative approach in the data presentation, but it turned out that the amount of recorded videos did not allow for statistically reasonable results and calculations based on images could be very misleading. Accordingly, the animal mobility does not fall into the category of mobile or immobile because they can move their legs with different frequency and also move their body with different frequency. This is why, we distinguished three levels of mobility (as presented in Figure 2). Moreover, the animal shape is only partially connected to animal mobility. As summarized in Figure 2, animals of croissant shape can move legs with different frequency. So we should mention that mobility of animals may result in differences in their appearance within a given experimental group that is visible in images. Thus, included videos truly reflect the animal state.

We tried to improve description of the toxicity test in the Material and Methods section as well as the Result section. We also corrected Fig. 2 to make it more understandable. The graphic was changed and some of the variants were transferred to Figure S1 (Additional file 1) just to show in Figure 2 the most important aspects of the test.

c. How the reversal of phenotype was tested? By replacing the medium with fresh medium without inhibitors or just wait and watch for 2 hours? If by wait and watch, then why reversal would be expected in the presence of inhibitors.

Answer: We agree with the Reviewer that this should be clearly described. We modified the proper part of the Materials and Methods section and Figure 2 legend to make the procedure understandable. The medium was not replaced till the end of the test. As far as the reversal is concerned, we assumed that due to increasing role of AOX, tardigrades are able to attenuate the mitochondrial respiratory chain inhibition with time. We address the issue in the Discussion section.

6. Page 7, lines 153-54: The statement…” the rapid disappearance of full activity was also observed when BHAM was added in the presence of AA applied in a sequence with KCN” is unclear.

Answer: AA was applied as a control for effectiveness of the MRC cytochrome pathway inhibition by KCN. To make it clear we corrected the relevant part of the manuscript, therefore the sentence was removed.

a. Was there no synergistic effect of AA with BHAM without KCN? If so, why?

Answer: AA is well known inhibitor of the MRC complex III. We did not applied AA alone in combination with BHAM as the MRC complex IV could still work (this problem could be omitted for isolated mitochondria). Instead, we used AA added in the presence of KCN to check if the inhibition of the MRC imposed by KCN is effective. Therefore, we introduced proper corrections to the description of the toxicity test to make it unequivocal. The same applies to measurements of the oxygen consumption rate.

b. From Figure S1, the morphology with AA treatment alone looks like that observed with BHAM.

Answer: Figure S1 presents the animals’ images at the beginning (30 min) and the end (2 h) of the toxicity test. At the end of the test the appearance of animals treated with AA and treated with BHAM was similar as the effects of AA and BHAM changed during the toxicity test and at the end of the test was similar. We introduced proper corrections to the description of the toxicity test as well as to the figure legend to make it unequivocal.

7. Figure 3:

a) Panel A) rates before and after each treatment should be given in the graph for easy comparison. Visually, BHAM’s effect is not apparent as described in the text.

Answer: The panel presented representative traces. The text mainly concerned the mean data presented in the panel B. We changed the figure and its description taking into account comments of both the Reviewers.

b) Panel B) Were the data obtained from experiments like in panel A? How many repeats were performed for the reported data in panel B? Is the reported data from one representative experiments or average of multiple experiments? How does it correlate with the data in Table 1? Is the reported data here based on 1st order or 2nd order kinetics?

Answer: The shown data were obtained in experiments like those shown in Panel A. As mentioned in the proper part of the Materials and Methods section (Measurements of *H. exemplaris* respiration), the measurements presented in Panel A were performed at least in triplicate. Panel B presented average of multiple experiments. Nevertheless, we changed Figure 3 taking into account comments of both the Reviewers. The changed version of Figure 3 includes information about repeats and statistical significance of the obtained results.

Table S1 (Additional file 3) presents oxygen consumption rates calculated in the presence or absence of animals as well as in the presence of the applied inhibitors. 1st order and 2^nd^ order denote order of KCN (±AA) and BHAM addition to the oxygraph chamber; BHAM after KCN ± AA and BHAM before KCN. We hope that the corrected version of the table is more reasonable.

c) Was the respiratory inhibition by BHAM alone significant? Show asterisks indicating significance level in panel B within the graph.

Answer: The respiration inhibition by BHAM alone was not statistically significant. We added the issue of statistical significance to the proper part of the Results section as well as to the figure legend. Importantly, the Figure 3 was changed and the statistical significance was added.

8. Table S1: Insufficient details is provided to understand this table.

a. Are the data in each row are from same experiment or different experiment? How many animals were used for obtaining these data? What is the respiration rate/animal? What is the degree of inhibition by each inhibitor individually and in combinations? Were the values reported for KCN, AA, and BHAM columns obtained independently or by sequential additions?

Answers: The data in each row are from the same experiment and represent calculations made for individual traces obtained in the presence and in the absence of animals as described in the Material and Methods section. The latter serve as a control for oxygen consumption. As indicated in Materials and Methods section, 10 000 animals were used for one trace. The presented values of oxygen consumption are calculated for 10 000 animals. The values reported for KCN, AA, and BHAM columns were obtained for independent experiments, represent values calculated for individual traces and do not reflect sequential additions. We corrected the table legend accordingly. We hope that the changed Figure 3 will help to understand Table 1 properly, including the impact of the inhibitors

b. It would be appropriate to show a graph superimposed with respiratory profile and the rates together for the sake of clarity.

Answer: The Figure 3 was changed accordingly.

c. Preferably, the respiration rate should be reported as the rate of oxygen consumption rather than the rate of uptake. Even though respiration rate will equal to the rate of uptake in principle, the uptake is not being measured directly. Oxygen consumption would be appropriate term as it is occurring to some degree even in the absence of animals in the chamber.

Answer: We agree with the Reviewer so we introduced the terminological change in the manuscript.

9. There is a mismatch in the functional data and AOX protein data supposed to detected by mass spectrometry only following 2 h treatment with KCN.

a. No efforts have been made to monitor the AOX protein or clone the gene using the information available to further characterize the AOX protein. It is possible that polyclonal antibody against AOX from other species can detect AOX of H. exemplaris. Alternatively, based on the protein sequence obtained from mass spectrometry and the sequences from other species, it would be possible to clone the gene by degenerate PCR, develop antibodies, compare, and characterize the AOX protein of H. exemplaris.

Answer: We agree with the Reviewer that it is worthwhile to work on specific antibody against *H. exemplaris* AOX. The predicted amino acid sequence of *H. exemplaris* AOX is available in NCBI data base but its C-terminus part is mis-annotaed (K. Arakawa personal communication). Unfortunately, our trials to verify the terminus sequence have not been successful yet. The antibody production is time taking procedure and at present is difficult to perform. We tried to detect the protein in whole-body extracts using antibody against plant AOX, but the result was not decisive that could be explained by low content of mitochondria and the AOX protein itself in the extracts. That is why we decided to apply mass spectrometry and it should be mentioned that the detection was possible only after animal treatment with KCN that indicates KCN-caused increase in AOX protein amount. We supplemented the mass spectrometry results with comparison of the detected peptides and amino acid sequence of *C. intestinalis* (Additional file 4).

b. The BHAM sensitivity alone is not enough to inform about H. exemplaris AOX function in mitochondrial bioenergetics. It is interesting to note that BHAM sensitivity is observed within 30-40 min (Fig. 3A) and the APX protein is detected following 2 h KCN treatment even with mass spectrometry, which is considered to be a highly sensitive technology. This discrepancy challenges the very basic foundation of this manuscript – that H. exemplaris AOX is function without the inhibition of Complexes III/IV.

**Answer:** We guess we were not clear enough with our conclusion. We do not discuss function of the enzyme in *H. exemplaris* bioenergetics as it requires further detailed studies. What we indicate is the possibility of the tardigrade *H. exemplaris* application to study AOX at animal organismal level because it is easy to observe the animal behaviour and perform respiration measurements of whole organisms. It is also possible to monitor the mitochondrial inner membrane potential in intact specimens by application of the specific fluorescence dye (TMRM) – we added the results to the revised manuscript. We also indicate issues that are interesting in further studies of the tardigrade AOX.

As far as the Reviewer comment to Figure 3A is concerned: this figure presented two types of oxygraphic records made for tardigrades to observe putative contribution of AOX to whole-body respiration. The effect of BHAM was not observed within 30-40 min, but was clearly seen after addition of KCN. Thus, the pronounced effect of BHAM on tardigrade respiration required the presence of the MRC cytochrome pathway inhibition imposed by the complex IV inhibitor. This observation does not exclude the presence of the protein earlier but it was possible to detect it after KCN treatment. Accordingly, it can be assumed that the AOX protein synthesis is increased when the cytochrome pathway is inhibited, allowing tardigrade cellular respiration when the main mitochondrial respiratory pathway is turned off. Interestingly, we observed a similar phenomenon during the measurements of AOX activity in respiration of the amoeba *Acanthamoeba castellanii* cells (unpublished data). We addressed the putative KCN contribution to AOX protein amount regulation in the Discussion section.

We agree with the Reviewer that mass spectrometry (SM) is a highly sensitive technology but there are some limits concerning detection of proteins with low level of expression. Since *H. exemplaris* AOX turned out to be expressed at the level enabling oxygraphic detection but not SM detection we decided to check if the inhibition of the cytochrome pathway could provide an increase in the protein amount. We only applied one duration of the animal treatment with KCN corresponding to the duration of the toxicity test, i.e. 2h. Of course it would be interesting to use different duration of the treatment and also apply different inhibitors of the mitochondrial respiratory chain but at present it is not possible due to the tardigrade limited culture.
